# Maternal Sleep Quality is Associated with Personal and Parenting Weight-Related Behaviors

**DOI:** 10.3390/ijerph17155312

**Published:** 2020-07-23

**Authors:** Kaitlyn M. Eck, Elena Santiago, Jennifer Martin-Biggers, Carol Byrd-Bredbenner

**Affiliations:** Department of Nutritional Science, Rutgers University, New Brunswick, NJ 08901, USA; elena.santiago@rutgers.edu (E.S.); jmartinbiggers@gmail.com (J.M.-B.); bredbenner@sebs.rutgers.edu (C.B.-B.)

**Keywords:** sleep, mothers, young children, weight-related behaviors, parenting practices

## Abstract

Mothers of young children tend to report poor-quality sleep, yet little is known about links between maternal sleep quality and weight-related behaviors and parenting practices. Thus, mothers of preschoolers completed an online cross-sectional survey assessing their sleep, physical activity, dietary behaviors, eating styles, child feeding practices, family meal behaviors, and health parameters. Comparisons by sleep quality using the Pittsburgh Sleep Quality Index item (i.e., very bad/bad, *n* = 87; fair, *n* = 255; and good/very good, *n* = 193) revealed mothers with poor-quality sleep had weight-related behaviors associated with higher Body Mass Index (BMI) (lower physical activity, fewer fruits/vegetables, more emotional and disinhibited eating). Poor-quality sleepers also engaged in parenting practices contrary to recommendations, such as less frequent modeling of healthy eating and physical activity, more control of child feeding, and fewer family meals. Mothers reporting poor-quality sleep tended to have lower parenting self-efficacy, poorer overall health status, more days of poor mental and physical health, greater depression, more stress, and higher BMIs. Future nutrition research should establish the directionality between sleep quality and health behaviors. Future interventions should help mothers develop strategies for improving sleep quality, such as increased physical activity and fruit and vegetable intake, and helping mothers realize how their sleep quality may affect parenting practices.

## 1. Introduction

It is widely recognized that healthy sleep is essential for good physical and mental health [1]. Characteristics of healthy sleep include being of adequate duration and quality [2]. Both sleep duration and quality can be adversely affected by lifestyle and environmental factors, including stressful schedules; erratic routines, such as those caused by shift work; poor diet; lack of physical activity; and excessive exposure to electronic devices, noise, and ambient light [3,4,5,6,7]. Sleep also can be affected by physiological factors, such as body weight, sleep apnea, and iron deficiency [8,9,10].

According to the National Sleep Foundation, the recommended sleep range for adults is 7 to 9 h per day [11]. However, many adults do not achieve this recommendation [11]. In 2012, 22% of the adults in the United States slept less than 6 h each night, which is a nearly one-third increase in prevalence from the prior three decades [12]. Shorter sleep duration is an independent risk factor for elevated health risks (e.g., weight gain and obesity) among individuals of all ages [13,14]. Compared to adults getting adequate sleep (between 7 to 9 h/night), those getting less sleep (≤6 h/night) are more likely to have a higher body mass index (BMI) [15,16,17]. Adults with shorter sleep duration also have an increased risk of cardiometabolic issues [18,19,20].

Sleep quality is a key factor interfering with achieving sleep duration recommendations [7,21]. Difficulty falling asleep and/or staying asleep impair sleep quality [11,21]. Mounting evidence indicates that sleep quality is associated with diminished health status [22]. For instance, poor quality of sleep is negatively related to mental health status (e.g., depression), functioning, and overall quality of life [23,24,25]. Poor sleep quality is also associated with an increased risk of cardiovascular disease, type 2 diabetes mellitus, and impaired glucose tolerance [23,24,25,26,27]. In addition, a study of twins discordant for BMI reported an inverse relationship between BMI and sleep quality [28].

Physical health conditions linked with poor sleep quality, including BMI, cardiovascular disease, and diabetes [10,29,30,31,32], also are linked to dietary intake and weight-status [33]. These shared links are likely because limited sleep duration is inversely correlated with physical activity, calorie intake, and overall diet quality, and is positively correlated with eating in response to negative emotions and availability of highly palatable foods (disinhibited eating) [34,35,36,37,38,39,40,41].

Sleep quality varies by sex, with women reporting significantly more sleep difficulties, or poorer sleep quality, than men [42,43,44]. Additionally, parenthood also affects sleep quality, with about a third to half of all parents of children reporting poor sleep quality [45,46]. Among parents, single parents, especially mothers, are more likely to report poorer sleep quality than adults without children (or with children over age 18 years) [46]. These findings suggest that mothers are an especially important group to study regarding sleep quality and its associations with maternal health behaviors. Further underscoring the importance of studying mothers is, in addition to the links between sleep quality and diet, maternal sleep quality may impact parenting behaviors related to child weight status [47].

Parenting behaviors associated with children’s weight status involve parental modeling of physical activity behaviors and attitudes toward physical activity [48,49,50] as well as the home food environment, including foods available, parent role modeling of eating behaviors, parent dietary intake, and family meal patterns [51,52,53,54,55,56,57]. Child feeding practices, such as restricting children’s access to palatable foods like sweets and snacks, also may adversely affect child weight [47,58,59]. Additionally, parents’ personal eating styles influence child feeding practices. For instance, parents who have a restrained eating style (i.e., restrict or regulate dietary intake to control weight) are more likely to restrict children’s access to snack foods, a behavior associated with potentially undesirable consequences [59]. Parents who lack confidence in their parenting abilities may be unable to perform behaviors protective of children’s weight status [60].

Although studies suggest poor sleep quality is associated with poorer overall dietary intake, attention to other weight-related behaviors remain understudied [37]. In addition, research focused on mothers with young children and variations in weight-related parenting behaviors by sleep quality could not be located. To the best of the authors’ knowledge, this is among the first studies to examine weight-related behaviors and parenting practices of mothers of young children vis-à-vis sleep quality. The researchers hypothesized that better sleep quality would be associated with healthier physical activity behaviors; dietary intake; eating styles; parenting practices, such as child feeding and family mealtime practices; household food availability; parenting self-efficacy; and health and weight status of mothers of preschool-aged children. This secondary data analysis of the Home Obesogenicity Measure of EnvironmentS (HOMES) survey, conducted from 2014 to 2016, aimed to increase understanding of the associations between sleep quality on weight-related personal and parenting behaviors of mothers with young children.

## 2. Materials and Methods

This research was approved by the Institutional Review Board at the authors’ university (protocol #11-294Mc; approved December 26, 2010 to present). All participants gave informed consent.

### 2.1. Participants

English-speaking mothers between the ages of 18 to 45 years old, who had one or more children between 2 to 5 years of age and were the primary household food purchaser and preparer were recruited to complete the HOMES study survey [61,62]. All who completed this online survey were recruited from the research panel of study participants from Survey Sampling International (SSI) and were compensated with points awarded by SSI that could be redeemed for gifts.

### 2.2. Instruments

The development of the HOMES survey is described in detail elsewhere [61,62,63,64]. In brief, the survey collected demographics, such as maternal race/ethnicity, highest education attained (high school or less, some post-secondary education, and baccalaureate college degree or higher), and age. Socioeconomic status was assessed with the Family Affluence Scale, a 4-item measure of family wealth. The FAS categorizes families as low, middle, or high affluence on a 10-point scale (0 to 9) and has shown good validity as a measure of socioeconomic status [65,66].

Weight-related measures that were assessed included sleep, physical activity, dietary behaviors, eating styles, child feeding practices, family meal behaviors, and health parameters. Sleep duration and quality were assessed using Pittsburgh Sleep Quality Index items [67,68]. Mothers’ reported total hours and minutes of sleep each night. Only mothers who reported biologically plausible sleep durations (between ±4 h of the mid-point of recommended sleep duration for adults [11,21], i.e., 4 to 12 h of sleep each night) were included in the analyses. Mothers also rated the overall quality of their sleep using a single 5-point Likert type item [67,68].

Mothers’ physical activity level was assessed using the HOMES Physical Activity Questionnaire [64,69]. The 3-item questionnaire measured the frequency mothers engaged in walking, moderate activity, and vigorous activity. An indicator item for sedentary behaviors assessed time (minutes/day) spent participating in screentime (i.e., watching TV or movies, playing games on computers or smart phones, and/or sending emails or text messages) daily. The frequency (days/week) that mothers modeled physical activity and sedentary behaviors was also measured.

Dietary behaviors assessed were daily intakes of sugar-sweetened beverages, milk, fruits/vegetables, and percent of calories from fat. The HOMES Drinks Intake Screener, a food frequency questionnaire, assessed servings/day of sugar-sweetened beverages (i.e., soft, fruit, tea, coffee, and energy drinks) and milk [70,71]. The 7-item Block Fruit/Vegetable/Fiber Screener, a food frequency instrument, measured daily servings of fruits and vegetables [72,73,74]. Percent of calories from fat was assessed with the Block Dietary Fat Screener [72,73,74], a food frequency instrument comprised of 17 items. Higher scores on these screeners indicate greater daily intake of sugar-sweetened drinks, milk, fruits/vegetables, and calories from fat, respectively.

Mothers’ eating styles were evaluated using the streamlined Emotional Eating, Adventurous Eating, Dietary Restraint, and Disinhibited Eating scales [62,75,76,77,78,79]. Higher scores on all these 4-point (definitely false, mostly false, mostly true, definitely true) Likert scales indicate greater expression of these eating behaviors. The Emotional Eating scale assesses how emotions influence the urge to eat or overeat. The Adventurous Eating scale evaluates the acceptance of new or unfamiliar foods. The Dietary Restraint evaluates one’s intention to restrict or regulate personal food intake to prevent weight gain. The Disinhibited Eating scale assesses temporary loss of control overeating behaviors.

Assessments of mothers’ use of non-recommended child feeding practices included control of children’s food intake choices, pressuring children to eat, and using food to encourage or reward children for eating healthy foods were included [54,62,80,81,82,83,84,85]. Higher scores on all these 5-point Likert (strongly agree to strongly disagree) child feeding practices scales indicate greater use of these child feeding practices by mothers. The Control of Child Intake scale determines the degree to which mothers exert control over the types of foods eaten by children. The Child Pressuring scale assesses whether mothers use verbal pressure to get children to eat healthy foods. The Feeding Rewards scale measures the use of palatable foods as a reward for eating healthy foods.

Other health-related parenting practices examined included maternal modeling of healthy eating and frequency of family meals and frequency of eating family meals in various locations (e.g., dining table, car, fast food restaurant), as well as household availability of fruits/vegetables, salty/fatty snacks, sugar-sweetened beverages, and milk. The Healthy Eating Modeling assesses how frequently mothers overtly model healthy eating behaviors to their children. Household food availability was assessed using food frequency screeners that evaluated the total servings available per household member per day. In addition, mother’s parenting self-efficacy (e.g., I feel sure about my parenting skills) [86] was evaluated using a 5-point Likert scale (not at all confident to very confident).

Health characteristics assessed included the overall health status and physical and mental health quality of life using the Centers for Disease Control and Prevention Health-Related Quality of Life measures [87,88]. Overall health status was assessed using a 5-point Likert scale (poor to excellent). Health quality of life is determined by the number of days per month the participant reports having poor mental and physical health. Maternal depression was assessed using the Patient Health Questionnaire-2 (PHQ-2) [89] and Cohen’s 4-item Perceived Stress Scale (PSS-4) was used to assess stress level [90]. The PHQ-2 and PSS-4 both assess the frequency of symptoms of depression and stress, respectively. Participants also reported heights and weights, which were used to calculate their BMI. Details for each study measure (e.g., total items, scoring, scale type, and Cronbach alpha) are summarized in Table 1.

### 2.3. Data Analysis

Mothers were partitioned into three groups based on sleep quality using the Pittsburgh Sleep Quality Index item: “During the past month, how would you rate our sleep quality overall?” The three groups were very bad/bad, fair, and good/very good. Spearman rank-order correlation was calculated to determine the relationship between sleep quality and duration. ANOVA and Tukey post-hoc tests were conducted to determine how physical activity, screentime, dietary intake, eating styles, child feeding practices, family meal practices, household food availability, parenting self-efficacy, and health differed by maternal sleep quality. To account for the multiple comparisons planned and reduce the risk of Type I errors, the Benjamini Hochberg procedure was employed at a rate of 5% for two-tailed tests. The statistical threshold for significance was set at *p* < 0.05 [91]. Analyses were performed with SPSS software version 26.0 (IBM Corporation, Chicago, IL, USA).

## 3. Results

Of the 910 participants who responded to the online survey recruitment, a total of 550 mothers met all study eligibility criteria and consented to participate. Of these, 15 were excluded due to biologically implausible sleep duration (i.e., <4 h/night or >12 h/night), thus the analytic sample equaled 535 mothers of young children. Mothers were an average age of 32.30 ± 5.81 SD years, with 72% indicating they were white and 28% non-white. Most (88%) mothers were in dual parent households. Most (83%) participants had at least some post-secondary education. Family affluence was moderate, averaging 5.63 ± 1.55 SD on the 0 to 9-point scale.

Nearly half of the mothers reported sleep quality was fair, with more than a third reporting good- or very good-quality sleep. One in eight mothers reported their sleep quality was very bad or bad. Single mothers (*n* = 64) reported significantly poorer quality sleep than the 471 in dual parent households (3.00 ± 0.98 vs. 3.27 ± 0.85, *p* = 0.04). ANOVA and Tukey post-hoc tests demonstrated that sleep quality and duration differed significantly for all pairwise comparisons (Table 1), with the lowest sleep quality group also having significantly shorter sleep duration than other groups. Sleep quality was significantly correlated with duration (Spearman R = 0.47, *p* = 0.001).

Maternal education and family affluence did not differ by sleep quality (*p* = 0.13; F = 2.03, *df* 2, 532; *p* = 0.26; F = 1.35, *df* 2, 532, respectively). A comparison of mothers’ physical activity level by sleep quality indicated that those in the poor-quality sleep group were significantly less physically active than those with good-quality sleep. Mothers with the worst sleep quality also modeled physical activity behaviors to children significantly less often than fair- and good-quality sleepers. Screentime, as an indicator of sedentary activity, and maternal modeling of sedentary screentime behavior did not differ by sleep quality.

Mothers in the poor-quality sleep group consumed significantly fewer servings of fruits and vegetables daily than good-quality sleepers. Sleep quality was not associated with intake of sugar-sweetened beverages, total fat, or milk. Few differences were noted in maternal eating styles. Those with good-quality sleep were significantly less likely to engage in emotional eating or disinhibited eating than fair-quality sleepers.

The good-quality sleep group had higher scores than comparison groups on the child feeding practices scales. Good-quality sleepers were significantly less likely to control their children’s food intake choices than mothers in the other groups. Good-quality sleepers also tended to use food rewards more than fair-quality sleepers. The good-quality sleep group modeled healthy eating more frequently, had more family meals, and served family meals more often at a dining table than mothers in the poor-quality sleep group. Mothers who reported good-quality sleep reported family meals were eaten at a fast food restaurant more days/week than both other groups. No differences were noted among sleep quality groups in the servings available per household member for fruits/vegetables, snack foods, sugar-sweetened beverages, or milk.

Mothers in the good-quality sleep group had significantly greater parenting self-efficacy scores than those with lower quality sleep. Mothers in the highest sleep quality group also reported significantly better health status than fair-quality sleepers, who, in turn, reported significantly better sleep quality than the low-quality sleep mothers. Mothers in the poor-quality sleep group reported significantly more days of poor mental or physical health each month and had higher depression severity and perceived stress scores than other mothers. Mothers in the good-quality sleep group had significantly lower BMIs than both other groups of mothers.

## 4. Discussion

Two-thirds of the sample of 535 mothers of young children reported having fair to very poor sleep quality, with single mothers having lower sleep quality than mothers in dual parent households. Sleep quality and duration were strongly positively correlated. The hypothesis that better sleep quality would be associated with healthier maternal physical activity behaviors, dietary intake, eating styles, parenting practices such as child feeding practices and family mealtimes, household food availability, parenting self-efficacy, and health and weight status was mostly borne out by the results. Mothers with poor-quality sleep had weight-related behaviors associated with higher BMIs, such as lower physical activity levels, fewer servings of fruits and vegetables, and more emotional and disinhibited eating. Poor-quality sleepers also tended to engage in weight-related parenting practices that run counter to recommendations, such as less frequent modeling of healthy eating and physical activity, more control of child feeding, fewer family meals overall, and fewer meals eaten at a dining table. However, mothers with good-quality sleep engaged in non-recommended practices, such as using food to reward children and having family meals at fast food restaurants. Mothers reporting poor-quality sleep also tended to have lower parenting self-efficacy, poorer overall health status, more days of poor mental and physical health, greater depression severity, more stress, and higher BMIs than comparators.

Women are more likely than men to experience poor sleep quality [92]. A recent study of women aged 20 to 76 years found that 38% had poor sleep quality [37], a rate more than double the proportion of women in this study reporting bad or very bad sleep. These incongruent results likely are due to differences in sleep assessment methodology, cut-off points used for sleep quality categorization, and sample characteristics, such as age and parenting responsibilities. For example, post-menopausal women tend to have poorer quality sleep than premenopausal women [93]. Compared to age-matched counterparts, mothers with young children tend to have poorer sleep quality, with caring for children being the most common reason women ages 25 to 34 years wake up during the night [94]. In addition, women’s perceptions of sleep sufficiency decline as the number of children under 18 in the home increases [42]. In the current study, single mothers reported significantly poorer sleep quality than mothers in dual parent households. This aligns with reports that women who slept with a romantic partner are significantly less likely to report daytime sleepiness or insomnia than other women [94], as well as findings that more single parents report inadequate sleep than other parents [42,46]. Further, single mothers are significantly less likely to wake up feeling rested and to have trouble staying asleep than mothers from two parent households [46]. As noted by others, another factor affecting sleep quality is socioeconomic status [95,96]. This may be a contributing factor to the poorer sleep quality of single mothers in this study in that single and dual parent mothers revealed differed significantly with regard to family affluence (i.e., 4.47 ± 1.58 SD vs. 5.79 ± 1.48, respectively).

Sleep quality impacts mental and physical health independently, and in combination [24]. The interconnectedness of sleep quality and quantity, as found in this study, can make it difficult to discern whether it is quality or quantity that is impacting health. The difficulty is further compounded when considering genetic factors affecting sleep duration [97,98,99]. Genetic variations suggest that high-quality relatively short sleep duration may be sufficient for some individuals supporting the importance of looking beyond sleep duration to considering the effect of sleep quality on health behaviors and outcomes. Sleep quality and sleep duration are independently associated with numerous measures of health. In a meta-analysis of 10 studies, poor sleep quality and short sleep duration independently increased the risk of type 2 diabetes [31]. Another meta-analysis revealed that poor sleep quality increased coronary heart disease risk [30] and that both long and short sleep duration elevated cardiovascular disease risk [30]. Sleeping less than the recommended 7 to 9 h per night increases the risk of obesity [100].

The relationship between sleep quality and physical activity is complicated by a bidirectional relationship. That is, physical activity improves sleep and better sleep quality leads to greater physical activity [101,102]. Sleep quality influences the physical activity level through its impact on psychological functioning (e.g., lower depression scores, positive coping strategies) [101,103]. Participation in exercise programs improve sleep quality in adults and physical activity is a beneficial treatment for depressive and anxiety disorders [104,105,106,107]. Although the cross-sectional design of the current study limits the ability to discern cause and effect, mothers in the lowest sleep quality group were significantly less physically active than mothers in the highest sleep quality group, displayed more depressive symptoms, and reported a significantly greater number of days of poor mental and physical health per month. The relationship between sleep duration and physical activity is mixed, with some researchers reporting that short duration sleepers are more physically active, possibly as a result of having more time to spend being active [100], whereas others report shorter sleep duration is associated with lower levels of physical activity [108]. In the current study, mothers in the highest sleep quality group were the most physically active and had the longest sleep duration. Further, the lack of differences in screen time between the sleep quality groups suggest that mothers in the lowest sleep quality group (shortest sleep duration) and highest BMI were spending their extra waking hours engaged in other sedentary activities.

When comparing maternal dietary intake by sleep quality, few significant differences were noted. Similar to other studies, poor sleep quality was associated with a lower intake of fruits and vegetables [109,110,111]. In prior research, poor sleep quality was associated with other measures of diet intake, including a greater intake of carbohydrates, particularly added sugar and sugar-sweetened beverages, and fat, especially saturated fatty acids [37,110,112,113]. However, the findings of the current study found no significant differences in sugar-sweetened beverage or dietary fat intake or in household availability by sleep quality. The varied methodology in assessing dietary intake likely explains these differing results. Improved sleep quality is commonly associated with improved diet quality [37,109] and St-Onge et al. suggest that dietary intake can impact sleep quality [114]. However, the cross-sectional designs of this and previous studies limit the ability to assign cause and effect. This study also did not assess the intake of food items, such as those containing caffeine, that may cause poor sleep.

Emotional eating and disinhibited eating were significantly lower in the highest sleep quality group. Dietary disinhibition mediates the relationship between poor sleep quality and body weight [33,40] and poor sleep quality is associated with increased emotional eating behaviors [115]. The relationship between sleep quality and eating behaviors is thought to be impacted by stress, which was demonstrated by this study’s findings. Research on the relationship between sleep quality and eating adventurousness is limited; however, short sleep duration is associated with less dietary variety [116]. In the present study, adventurous eating did not differ by sleep quality.

Little is known about sleep quality or duration and parenting practices relationships. Two studies reported that parents with poor sleep quality were more likely to utilize undesirable permissive parenting behaviors [117,118]. In the current study, mothers with the best sleep quality vs. those with poorer sleep quality had a greater tendency to utilize both recommended (less control of child intake) and, contrary to the study hypothesis, non-recommended (rewarding children with food) child feeding practices. The non-recommended practice of pressuring children to eat did not differ by sleep quality, perhaps because pressuring behaviors tends to differ by child gender, which was not considered in this research [119]. Maternal modeling of healthy eating behaviors also did not differ by sleep quality, which aligns with the few differences in maternal dietary intake by sleep quality. Mothers with better sleep quality had the greatest frequency of family meals. Although these high sleep quality sleepers had more family meals at fast food restaurants than counterparts, the total proportion of meals high-quality sleepers ate in locations associated with lower nutritional quality [120,121] (i.e., in a car, at a fast food restaurant, and in front of the television) accounted for only 23% of total family meals compared to 26% and 29% for fair- and low-quality sleepers, respectively. Mothers in the high sleep quality group may have chosen to reward children with food as well as serve fast food as a form of stress and/or time management, which allowed them to spend more time sleeping and have higher quality sleep [122]. As noted in this study, mothers in the highest sleep quality group had significantly lower perceived stress and greater stress management (felt they were better able to control stress) than mothers in the other groups.

Mothers reporting poor-quality sleep also tended to have poorer overall mental and physical health status and higher BMIs than other mothers, which is in congruence with other studies demonstrating a causal relationship between poor sleep quality and poor mental health outcomes, including anxiety, depression, and stress [123,124,125]. The independent links between both sleep quality and sleep duration and obesity are also well established [32,126,127]. Higher body weights associated with poor sleep quality may be the result of decreased physical activity, poorer diet quality, and/or increased emotional and disinhibited eating [33,37,40,101,102,109,115].

Previous studies have focused primarily on the relationships between sleep duration and health. An important strength of this study was the exploration of the relationship between sleep quality and mothers’ own weight-related behaviors. In addition, this is among the first studies to examine weight-related parenting behaviors vis-à-vis sleep quality. This study had numerous other strengths, including the large sample size of mothers who were diverse in race/ethnicity and lived in geographically diverse regions distributed throughout the United States. Additionally, the survey was comprised of valid reliable measures. Study strengths must be considered in light of limitations. This study is limited by its self-report nature as well as the relatively high socioeconomic status and focus on females. Further, this study’s cross-sectional design inhibits the ability to discern cause and effect as well as the directionality of the relationship between sleep quality and weight-related behaviors [32,126,127]. The possible effect of total children in the household on sleep quality and/or child sex on parenting behaviors and sleep also were not considered [42,59]. To expand the understanding of sleep quality and weight-related behaviors of mothers, it would be worthwhile for future research studies to include a more diverse sample (e.g., socioeconomic status, family size). Additionally, future research studies should investigate how mothers’ sleep quality varies over the course of the family lifecycle to ascertain how sleep quality varies as children grow and mature and how changing family circumstances (e.g., divorce, employment changes) link with sleep hygiene.

## 5. Conclusions

Better sleep quality was found to be associated with healthier maternal physical activity behaviors, eating styles, parenting self-efficacy, and health and weight status of mothers of preschool-aged children as was hypothesized. The directionality of the links observed between sleep quality and healthier behaviors could not be determined by this cross-sectional study; however, research suggests these relationships may be synergistic and bidirectional [128,129,130]. Another key finding that sleep quality had little to no association with maternal diet quality and household food availability is contrary to findings from other studies [37,104]. Future research should endeavor to investigate how the constellation of weight-related behaviors interact and affect sleep quality. In addition, future nutrition education and weight-management interventions aiming to improve health in mothers and their young children may be improved by providing mothers with opportunities to develop strategies for improving behaviors that could lead to better sleep quality, such as increased physical activity level and fruit and vegetable intake, while also helping mothers realize how their own sleep quality may be affecting parenting decisions and interactions with children.

## Figures and Tables

**Table 1:** Weight-related behaviors of mothers of young children by maternal sleep quality (*n* = 535).

Measure	Number of Items	Possible Score Range	Cronbach Alpha	Sleep Quality Rating			F (df = 2, 532); Main Effects *p*-Value	Tukey Post Hoc Tests **
				Very Bad/Bad *n* = 87	Fair *n* = 255	Good/Very Good *n* = 193		
				Mean ± SD (Lower, Upper 95% CI)	Mean ± SD (Lower, Upper 95% CI)	Mean ± SD (Lower, Upper 95% CI)		
**Maternal Sleep**								
Sleep Duration (hours/day)	1	0 to 24	*	5.99 ± 1.18 (5.73, 6.23)	7.00 ± 1.09 (6.87 ± 7.14)	7.66 ± 1.08 (7.51, 7.82)	70.74; <0.0001	ABC
Sleep Quality ^A^	1	1 to 5		1.84 ± 0.37 (1.76, 1.92)	3.00 ± 0.00 (3.00, 3.00)	4.19 ± 0.40 4.14, 4.25)	2300.49; <0.0001	ABC
**Maternal Physical Activity**								
Physical Activity Level ^B^	3	0 to 42	*	12.34 ± 9.63 (10.29, 14.40)	15.11 ± 9.85 (13.89, 16.32)	16.55 ± 9.68 (15.18, 17.92)	5.59; 0.004	B
Screentime (minutes/day)	1	0 to 1440	*	369.48 ± 278.69 (310.09, 428.22)	405.94 ± 315.45 (367.04, 444.84)	347.80 ± 272.61 (309.09, 386.50)	2.19; 0.113	
Physical Activity Modeling (days/week)	1	0–7	*	2.73 ± 1.18 (2.48, 2.98)	3.11 ± 1.27 (2.95, 3.26)	3.17 ± 1.16 (3.00, 3.33)	4.10; 0.017	AB
Screentime Modeling (days/week)	1	0–7	*	2.76 ± 2.37 (2.26, 3.27)	2.77 ± 2.11 (2.50, 3.03)	2.93 ± 2.20 (2.62, 3.24)	0.342; 0.710	
**Maternal Dietary Intake**								
Sugar-Sweetened Beverage Intake ^C^ (servings/day)	4	0 to 4.6	*	0.46 ± 0.50 (0.35, 0.57)	0.58 ± 0.49 (0.52, 0.64)	0.60 ± 0.49 (0.53, 0.67)	2.60; 0.075	
Fruit and Vegetable Intake ^D^ (servings/day)	7	0 to 12.17	*	4.05 ± 1.73 (3.68,4.42)	4.48 ± 2.19 (4.21, 4.75)	4.73 ± 2.31 (4.40, 5.05)	2.95; 0.053	
% Calories from Total Fat ^E^	17	0 to 100	*	37.23 ± 5.21 (36.12, 38.34)	37.32 ± 5.43 (36.65, 37.99)	37.07 ± 6.21 (36.19, 37.95)	0.10; 0.901	
Milk ^C^ (servings/day)	1	0 to 8	*	3.77 ± 3.19 (3.09, 4.45)	3.80 ± 3.04 (3.43, 4.18)	4.11 ± 3.08 (3.67, 4.55)	0.647; 0.524	
**Maternal Eating Styles**								
Emotional Eating ^F^	3	1 to 4	0.75	2.12 ± 0.89 (1.93, 2.31)	2.18 ± 0.89 (2.07, 2.29)	1.88 ± 0.82 (1.76, 1.99)	6.74; 0.001	C
Adventurous Eating ^F^	2	1 to 4	0.72	3.07 ± 0.67 (2.93, 3.21)	3.18 ± 0.70 (3.09, 3.26)	3.19 ± 0.66 (3.10, 3.29)	1.07; 0.343	
Dietary Restraint ^F^	4	1 to 4	0.74	2.38 ± 0.77 (2.22, 2.55)	2.41 ± 0.72 (2.32, 2.50)	2.44 ± 0.74 (2.34, 2.55)	0.26; 0.775	
Disinhibited Eating ^F^	3	1 to 4	0.81	2.03 ± 0.71 (1.88, 2.18)	2.01 ± 0.77 (1.91, 2.10)	1.83 ± 0.73 (1.73, 1.94)	3.58; 0.028	C
**Child Feeding Practices**								
Control of Child Food Intake ^G^	3	1 to 5	0.61	2.50 ± 0.91 (2.31-2.69)	2.43 ± 0.89 (2.32-2.54)	2.22 ± 0.89 (2.09-2.34)	4.36; 0.013	BC
Child Pressuring ^G^	3	1 to 5	0.69	1.99 ± 0.83 (1.81, 2.17)	2.13 ± 0.88 (2.02, 2.23)	2.23 ± 1.04 (2.09, 2.38)	2.12; 0.121	
Feeding Rewards ^G^	3	1 to 5	0.73	2.50 ± 0.85 (2.32, 2.68)	2.54 ± 0.83 (44, 2.64)	2.76 ± 0.96 (2.62, 2.90)	4.29; 0.014	C
Healthy Eating Modeling ^G^	4	1 to 5	0.56	3.38 ± 0.68 (3.24, 3.52)	3.47 ± 0.74 (3.38, 3.56)	3.62 ± 0.74 (3.51, 3.72)	3.88; 0.021	B
**Family Meal Behaviors**								
Family Meal Frequency (meals/week)	1	0–21	*	12.16 ± 5.40 (11.01, 13.31)	13.49 ± 5.03 (12.87, 14.11)	14.30 ± 4.83 (13.61, 14.99)	5.50; 0.004	B
**Family Meal Location (days/week)**								
Dining Table	1	0–7	*	4.22 ± 2.61 (3.66, 4.78)	4.55 ± 2.60 (4.23, 4.87)	5.04 ± 2.29 (4.72, 5.37)	3.86; 0.022	B
Car	1	0–7	*	0.33 ± 0.97 (0.13, 0.54)	0.32 ± 0.83 (0.22, 0.42)	0.48 ± 1.34 (0.29, 0.67)	1.41; 0.245	
Fast Food Restaurant	1	0–7	*	0.71 ± 0.93 (0.52, 0.91)	0.80 ± 0.90 (0.69, 0.91)	1.06 ± 1.32 (0.87, 1.24)	4.48; 0.012	BC
In Front of TV	1	0–7	*	2.53 ± 2.57 (1.98, 3.08)	2.36 ± 2.49 (2.05, 2.67)	1.82 ± 2.33 (1.49, 2.15)	3.62; 0.028	
**Household Food Availability**								
Fruits/Vegetables ^C^	7	0–8		3.07 ± 1.55 (2.74, 3.40)	3.39 ± 1.40 (3.22, 3.57)	3.37 ± 1.45 (3.17, 3.58)	1.75; 0.176	
Salty/Fatty Snack Foods ^C^	4	0–32	*	7.15 ± 5.66 (5.94, 8.35)	7.98 ± 6.72 (7.15, 8.81)	8.84 ± 7.72 (7.74, 9.93)	1.93; 0.146	
Sugar-Sweetened Beverages ^C^	4	0–8	*	1.57 ± 0.98 (1.36, 1.79)	1.58 ± 1.23 (1.43, 1.73)	1.82 ± 1.41 (1.62, 2.03)	2.39; 0.101	
Milk ^C^	1	0–8	*	3.15 ± 3.13 (2.48, 3.82)	2.81 ± 2.93 (2.45, 3.17)	2.74 ± 2.70 (2.36, 3.12)	0.627; 0.535	
**Parenting Self-Efficacy** ^H^	1	1–5	*	3.89 ± 0.88 (3.70, 4.07)	4.00 ± 0.82 (3.90, 4.10)	4.30 ± 0.71 (4.19, 4.40)	10.96; <0.0001	BC
**Maternal Health**								
General Health Rating ^I^	1	1 to 5	*	3.18 ± 0.90 (2.99, 3.37)	3.43 ± 0.85 (3.33, 3.54)	3.80 ± 0.79 (3.69, 3.91)	19.18; <0.0001	ABC
Physical Health-related Quality of Life (unhealthy days/month)	1	0 to 30	*	4.86 ± 6.89 (3.39, 6.33)	2.27 ± 4.22 (1.75, 2.79)	1.82 ± 3.76 (1.28, 2.35)	13.81; <0.0001	AB
Mental health-related Quality of Life (unhealthy days/month)	1	0 to 30	*	7.64 ± 8.71 (5.79, 9.50)	4.35 ± 7.54 (3.42, 5.28)	2.03 ± 4.65 (1.37, 2.69)	20.53; <0.0001	ABC
Patient Health Questionnaire-2 (Depression Severity) ^J^	2	1 to 4	0.81	1.64 ± 1.59 (1.30, 1.98)	1.03 ± 1.40 (0.86, 1.20)	0.75 ± 1.25 (0.57, 0.93)	12.53; <0.0001	AB
Perceived Stress ^J^	4	1 to 4	0.69	2.24 ± 0.768 (2.08, 2.40)	1.97 ± 0.66 (1.89, 2.05)	1.69 ± 0.60 (1.60, 1.77)	23.10; <0.0001	ABC
Body Mass Index (BMI)	1	2	*	29.04 ± 8.60 (27.20, 30.87)	28.51 ± 8.13 (27.50, 29.51)	26.08 ± 7.03 (25.08, 27.08)	6.76; 0.001	BC

* Cronbach’s alpha is not applicable. ** Tukey post-hoc tests: A = significant difference between low and moderate sleep quality; B = significant difference between low and high sleep quality; C = significant difference between moderate and high sleep quality. ^A^ 5-point rating scale: very bad, bad, okay, good, very good; scored 1 to 5 respectively with higher scores indicate better sleep quality. ^B^ 8-point Exercise Days/week: 0, 1, 2, 3, 4, 5, 6, and 7; days/week weighted by exercise intensity (weights of 1, 2, 3 for walking, moderate, and vigorous activity, respectively) and summed to create scale score; higher scale score indicates greater activity level. ^C^ 9-point Servings Rating: <1 time/week, 1 day/week, 2 days/week, 3 days/week, 4 days/week, 5 days/week, 6 days/week, 7 days/week, >1 time/day; scored 0 to 8 respectively. ^D^ 6-point Servings Rating: <1 serving/week, 1 serving/week, 2 to 3 servings/week, 4 to 6 servings/week, 1 serving/day, 2 or more servings/day; scored 0 to 5 respectively; scale scoring algorithm is protected by copyright and described in detail elsewhere [72]. ^E^ 5-point Servings Rating: 1 time/month or less, 2 to 3 times/month, 1 to 2 times/week, 3 to 4 times/week, 5 or more times/week; scored 0 to 4, respectively; scale scoring algorithm is protected by copyright and described in detail elsewhere [72]. ^F^ 4-point Agreement Rating: definitely false, mostly false, mostly true, definitely true; scored 1 to 4, respectively. Items averaged to create scale score; higher scores indicate greater expression of the trait. ^G^ 5-point Agreement Rating: strongly disagree, disagree, neither agree nor disagree, agree, strongly agree; scored 1 to 5 respectively; scale score equals average of item scores with higher scale score indicating greater expression of the trait. ^H^ 5-point Confidence Rating: not at all confident, not confident, confident, quite confident, very confident; scored 1 to 5 respectively; higher scale score indicates greater confidence. ^I^ 5-point Excellence Rating: poor, fair, good, very good, excellent; scored 1 to 5 respectively; higher score indicates better health. Possible score range = 1 to 5. ^J^ 4-point Occurrence Rating: not at all, several days, more than half the days, nearly every day; scored 1 to 4, respectively; scale score equals average of items; higher scores indicate greater expression of the behavior.
